# Feelings, Thoughts, and Behaviors During Disaster

**DOI:** 10.1177/1049732320968791

**Published:** 2020-11-23

**Authors:** Alessandro Massazza, Chris R. Brewin, Helene Joffe

**Affiliations:** 1 Research Department of Clinical, Educational and Health Psychology, University College London, London, United Kingdom

**Keywords:** peritraumatic, trauma, PTSD, disaster, earthquake, 2016-2017 Central Italy earthquakes, qualitative, Italy

## Abstract

Thoughts, feelings, and behaviors during traumatic events, that is, peritraumatic reactions, are key to post-trauma psychopathology development. Qualitative research is required to investigate whether existing quantitative methods capture the range and complexity of peritraumatic reactions as described by survivors. Semi-structured interviews were conducted with 104 earthquake survivors. Participants reported experiencing various peritraumatic reactions (*M* = 21, range = 6–43). The survivors’ accounts confirmed presence and overall phenomenological characteristics of commonly studied peritraumatic reactions such as dissociation, distress, mental defeat, and immobility. In addition, novel and understudied reactions were identified: cognitive overload, hyperfocus, and emotion regulation, as well as positive affect. Finally, a number of cross-cutting phenomena were identified such as the social nature of many reactions and survivors evaluating their reactions as difficult to put into words. These findings have implications for the conceptualization of peritraumatic reactions, for trauma-focused psychotherapeutic interventions, and for the wellbeing of disaster survivors.

The field of trauma and post-traumatic stress disorder (PTSD^1^) has highlighted the thoughts, feelings, and behaviors people experience during or immediately after exposure to traumatic events (Bovin & Marx, 2011). These phenomena have been collectively named “peritraumatic reactions.” From Janet’s studies on dissociation in hysteria onwards (Janet, 1889), peritraumatic reactions have occupied a central role in defining the experience of trauma and post-trauma psychopathology (van der Kolk & Fisler, 1995). The most influential theories on the development of PTSD suggest that what happens at the time of the traumatic event and its consequences for the encoding of traumatic memories is key to understanding the condition (Brewin et al., 1996; Ehlers & Clark, 2000).

A large volume of literature has been devoted to understanding the link between peritraumatic reactions and post-trauma psychopathology (Gorman et al., 2016). The systematic study of peritraumatic reactions began with Marmar et al.’s (1994) study of peritraumatic dissociation at the end of the 1990s and with the inclusion of Criterion A2 (i.e., traumatic event had to be accompanied by “intense fear, helplessness, and horror”) in the *Diagnostic and Statistical Manual of Mental Disorders* (4th ed.; *DSM-IV*; American Psychiatric Association, 1994) PTSD diagnosis in 1994. Since then, the literature on peritraumatic phenomena has been growing steadily with more than 800 papers published on the topic each year in the past few years.^2^ Meta-analytic studies of risk factors for PTSD prior, during, and following the traumatic event have identified peritraumatic reactions as some of the most important risk factors for PTSD development (Brewin et al., 2000; Ozer et al., 2003).

The reactions that have received the most attention are peritraumatic dissociation, both in its psychic (Candel & Merckelbach, 2004; Nobakht et al., 2019) and somatoform (Nijenhuis, 2004) presentations, distress (Brunet et al., 2001; Kannis-Dymand et al., 2019), tonic immobility (Hagenaars & Hagenaars, 2020; Marx et al., 2008), panic attacks (Nixon & Bryant, 2003), data-driven processing (Halligan et al., 2002), and mental defeat (Dunmore et al., 2001). While these reactions are believed to be somewhat phenomenologically distinct from one another (Massazza et al., 2020), they tend to be associated with higher levels of post-trauma psychopathology, most commonly PTSD (Vance et al., 2018), but also depression (Bunnell et al., 2018).

The existing work on peritraumatic phenomena has based the identification of different peritraumatic reactions mainly on accounts from clinical experience (e.g., dissociation), and insights from animal models (e.g., tonic immobility) or psychological theory (e.g., data-driven processing). While these are reasonable methods to determine the presence of such reactions, inductive, systematic qualitative work is needed to provide a naturalistic and scientifically accurate account of the lived experience of peritraumatic reactions among trauma survivors (Tatano Beck, 2011).

In addition, the current approach to studying peritraumatic reactions is largely based on the use of standardized questionnaires such as the Peritraumatic Dissociative Experiences Questionnaire (Marmar et al., 1994) and the Peritraumatic Distress Inventory (Brunet et al., 2001). This deductive method is inevitably bound to confirm the presence of these reactions by enquiring specifically about them, possibly missing important, clinically relevant experiences. Indeed, when more inductive qualitative approaches have been used to explore survivors’ recollections of reactions during traumatic events, the findings have suggested a more nuanced and complex range of experiences to those identified in the peritraumatic literature.

In the largest qualitative study to use interviews to investigate spontaneously reported reactions during traumatic events, 125 survivors of various disasters across different European countries were asked to recall in a free narrative their own reactions (Grimm et al., 2014). Across the sample, the most frequently reported emotions and cognitions were fear, panic (as in amplification of “fear” rather than mass panic or panic attacks), and perceptions of high risk. Conversely, the most frequently reported behaviors were extending support to others and attempting to save lives. Qualitative studies among survivors of various emergencies also provide evidence for survivors spontaneously comforting and supporting or cooperating with each other during traumatic events (Drury, 2018; Wilson et al., 2012). Furthermore, approximately half of the sample reported that they reacted in a calm and rational manner, while the other half reported acting at an instinctual and automatic level. A minority described not reacting at all due to resignation. Other reactions that were identified included detachment, relief, emotion regulation, nervousness, dissociation, seeking information, and preparing for evacuation.

However the study was limited by a considerable time lag between the events and the retrospective accounts, that is, an average of 4 years, by the lack of homogeneity between the different traumatic events among participants, and by most interviews being conducted in focus groups, which are not ideal settings for openly discussing some peritraumatic reactions (e.g., guilt, shame) due to social desirability bias.

Qualitative studies examining peritraumatic reactions specifically during earthquakes have also confirmed a more diverse range of reactions than those identified in the psychotraumatology literature (Kannis-Dymand et al., 2015; O’Toole, 2017; Prati et al., 2012). In a qualitative study among teachers (*N* = 20) during the 2011 Christchurch earthquake, many engaged in various emotional regulation techniques such as self-talk and deep-breathing, and problem-focused coping to give an impression of calm while prioritizing the wellbeing of their students (O’Toole, 2017). These emotional reduction and disengagement mechanisms have received some attention in the first-responders literature (Hammock et al., 2019; Levy-Gigi et al., 2016), but have not been addressed in peritraumatic work.

The current focus of the psychotraumatology literature on a small subset of negative peritraumatic reactions therefore appears incomplete when compared with the more nuanced and complex picture provided by experiential accounts of disaster survivors. The current study will attempt to address these limitations and provide a naturalistic account of the lived experience of peritraumatic reactions by taking a more inductive, qualitative approach and asking participants to spontaneously report feelings, thoughts, and behaviors experienced during key distressing moments of the same traumatic event.

## Method

### Participants and Recruitment

All participants were survivors of the 2016-2017 Central Italy earthquakes. A purposive sampling strategy was used to identify a sample that reflected the demographic distribution of the population as a whole in terms of gender and age as per the 2016 census data (Istituto Nazionale di Statistica, 2016). The recruitment process was aided by the local health center and municipality. Participants were contacted either by phone or approached face to face. In addition, participants who had participated in a previous study (Massazza et al., 2019) were reinvited to participate in the current wave of data collection. The recruitment was aided by the trusting relationship the authors had built with the population during previous research, which has been highlighted as an important basis of access in rural areas (Hamilton, 2020).

### Procedure

Semi-structured interviews were conducted in Italian with 104 survivors. Participants were asked to identify key distressing moments they had experienced as part of the earthquake’s sequence. They were then presented with the following standard prompt:

I would now like you to try and go back in memory to the moments of the events that correspond to the memory you have just described [refer to the moments described in the memory] and try and describe in as much detail as you can what you felt, thought and did in those moments. It is important that you think back to what happened in the exact moments described by the memory.

At the end of the interview, the additional prompt was presented: “Is there anything else you did, thought or felt?.” The interviewer did not use an interview guide or inject any external content related to preidentified peritraumatic reactions to allow the participant’s narrative to form freely and naturalistically. Interviews lasted an average of 1 hour and were tape-recorded.

The use of interviews was deemed necessary to address our research questions as the use of standard peritraumatic surveys would have only allowed for a number of predefined peritraumatic reactions to emerge and would have not provided detailed phenomenological data on each reaction (but see Massazza et al., 2020 for a study on the same population using standard peritraumatic measures).

Data collection took place across 3 months in May, June, and July 2018. This was 20 months following the August 2016 earthquake and 15 months after the January 2017 earthquake. The UCL Research Ethics Committee approved a larger mixed-methods study of which this project was a subcomponent with the project ID: 10517/001. Prior to taking part, participants read an information sheet and provided written informed consent. Participants were made aware of their right to withdraw from the study at any point, although no participant expressed a desire to do so.

### Data Analysis

All 104 interviews were transcribed verbatim in Italian. We followed the methodological framework for thematic analysis as described by Joffe (2012). While transcribing, recurring peritraumatic reactions were noted down to create a preliminary thematic framework. The various codes corresponding to different peritraumatic reactions were then clustered into thematically related groups of codes to facilitate the analysis. The assignment of codes to particular groups/clusters followed from discussions among the authors and from inspection of code co-occurrence tables to assess which codes spontaneously appeared most in association with other codes (Contreras, 2011).

Following the methodological framework described by Fereday and Muir-Cochrane (2006), we used a hybrid approach encompassing a largely inductive framework with the inclusion of some theory-driven constructs (e.g., dissociation, mental defeat, cognitive overload). This was justified by the fact that while we were interested in letting new themes emerge spontaneously from the participants’ narratives, we also wanted to explore whether and how commonly researched peritraumatic reactions would have been reported by participants when unprompted. However, we tried to remain as close as possible to the specific wording used by participants whenever appropriate (e.g., only coding for “panic” when participant specifically spoke of “panic”).

To assess the reliability of the coding frame, the authors explained the framework to a second coder who was naïve to the field of peritraumatic reactions and blindly coded approximately 5% of the entire data set (O’Connor & Joffe, 2020). A substantial intercoder reliability rate was achieved between coders, with an average kappa of .73. Discrepancies were discussed and resolved. Following discussion among the authors, the thematic framework was finalized. Distressing moments were accepted only if they had happened between the moment of the earthquake shock on the 24th of August and the State funerals that took place on the 30th of August or during the earthquake shocks of the 30th of October and 18th of January as per definition of peritraumatic, that is, occurring during or immediately after the traumatic event (Gorman et al., 2016). All sections corresponding to the peritraumatic time frame were then thematically analyzed to explore the most frequent themes in depth.

Our thematic analysis was grounded in a phenomenological epistemological framework as we set out to analyze and report on the experiences, meanings, and reality as discussed by the participants themselves to represent the lived experiences of these reactions. While all authors had expertise in the field of peritraumatic reactions, we attempted to approach the data without preconceived assumptions and remain as close as possible to the specific wording used by participants. The substantial intercoder reliability rate achieved with a second coder naïve to the field of peritraumatic reactions is reassuring in this respect. The analysis was conducted using ATLAS.ti (version 7).

## Results

### Participant Demographics

Demographic information on the current sample is provided in Table A in Supplemental Materials.

### Description of Distressing Moments

Participants identified various key distressing moments during the semi-structured interviews. The most commonly reported events are presented in Table 1. A number of key distressing moments, and their corresponding peritraumatic reactions, were excluded from the thematic analysis as they took place outside of the peritraumatic time frame.

**Table 1.** Content of Key Distressing Moments Identified by Participants, in Order of Prevalence.

Content	Number of Times Mentioned
Earthquake shock on the 24th of August	32
Seeing corpses or body parts in the debris	20
Collapsing buildings and material devastation	16
Rescue of individuals from debris	12
Recognition and management of corpses	11
Moments of realization of death of people	10
Finding out/being told of death of people	8
State funerals and/or private funerals	6
Earthquake shock on the 30th of October	6
Witnessing people in distress	4
Getting out of collapsing buildings	4
Being stuck under debris	4
Earthquake shock on the 18th of January	4

*Note.* Distressing moments mentioned less than 4 times are not included in this table.

### Description of Peritraumatic Reactions

A total of 85 different peritraumatic reactions were identified in the interviews (see Table 2). Each code was assigned to one of the seven groups of codes: emotional distress; action, hyperfocus, and emotion regulation; cognitive overload; dissociation; mental defeat and loss of control; immobility and somatic reactions; and positive affect. Some codes could have belonged to various groups and were therefore placed in the group to which they most aligned according to how they were discussed by participants. Most participants (81%) also described the reactions of other people around them during interviews. In addition, more than half of the participants (53%) mentioned struggling to describe certain reactions in their own words and 25% of participant described their reactions as “strange.” Finally, 19% of participants reported experiencing several different, at times contradictory, reactions at the same time. Each participant reported experiencing a mean of 21 different reactions at the time of the traumatic event (range = 6–43), indicating considerable fluctuations between different types of peritraumatic reactions. Due to the volume of peritraumatic reactions identified, only those spontaneously reported by at least 25% of the sample are described qualitatively below, but all are mentioned in Table 2. Each group of peritraumatic codes will be presented separately, in order of prevalence.

**Table 2.** Descriptive Information on Codes Under Each Code Group in Order of Prevalence.

1. Emotional Distress	2. Action, Hyperfocus, and Emotion Regulation	3. Cognitive Overload
Fear for others (78%)	Gaining awareness (78%)	Confusion (63%)
Fear (70%)	Goal-oriented actions (72%)	Not knowing what to do (46%)
Uncertainty (63%)	Trying to be useful/Helping others (64%)	Overwhelmed/shocked (45%)
Sadness, emotional pain (43%)	Staying calm (38%)	Not realizing the extent of damage (30%)
Anger (35%)	Urge to act/Being reactive (34%)	Feeling lost (24%)
Urge to flee (34%)	Maintaining clarity of thoughts/self-control (32%)	Having no thoughts, thought vacuum (22%)
Anxiety about future (32%)	Calming others/Providing emotional support (31%)	Racing thoughts (14%)
Catastrophic thinking (30%)	Being strong/courageous (25%)	Geographical disorientation (11%)
Anxiety (29%)	Instinctual behaviors (25%)	Disorganized thoughts (10%)
Thought of death (26%)	Concentrated/Focused (21%)	
Crying (22%)	Feeling prepared (21%)	
Guilt/ Shame (20%)	Taking leadership/Taking initiative (20%)	
Feeling trapped (19%)	Detachment (18%)	
Panic (17%)	Being strong for other people (17%)	
Screaming (16%)	Heightened physical capacities (17%)	
Desperation (13%)	Sense of duty (11%)	
Why questions (13%)	Trying to shield other people from horror (10%)	
Guilt for having survived (4%)	Earthquake survival strategy (9%)	
4. Dissociation	5. Mental Defeat and Loss of Control	6. Immobility and Somatic Reactions
Distortions in sense of reality (51%)	Helplessness (60%)	Physical immobility (37%)
Unbelievability (43%)	Feeling defeated (30%)	Feeling cold (15%)
Numbness (26%)	Feeling useless/insignificant/defenseless (26%)	Unusual body experiences (15%)
Distortions in sense of time (21%)	Feeling vulnerable/lonely (16%)	Not feeling pain (i.e., anesthesia; 11%)
Distortions in sense of self (20%)	Exhaustion (15%)	Loss of appetite and thirst (10%)
Feeling of void (13%)	Lack of control (14%)	Fainting (9%)
Feeling like on automatic pilot (11%)	Loss of emotional control/Feeling “crazy” (14%)	Difficulty breathing (8%)
Feeling like a spectator (9%)	Feeling like something else has control of body (3%)	Shaking (7%)
Failing to notice obvious things (5%)		Fear-related bodily sensations (4%)
		Feeling like throwing up/Throwing up (3%)
		Loss of control over bowel or bladder (2%)
7. Positive Affect		
Hope (29%)		
Joy (24%)		
Social connectedness (21%)		
Sense of invincibility/exaltation (10%)		
Emotional liberation (5%)		
Awe (2%)		

*Note.* The number in brackets corresponds to the percentage of the total sample that reported experiencing the reaction.^3^ The code groups are presented in order of prevalence, calculated by summing together the percentages of each code within each group.

Certain reactions did not belong to any specific group such as “appealing to God” or “religious coping” (18% of the total sample), “over-identification with other people” (15%), “avoiding distressing scenes” (14%), “being in physical pain” (7%), “feeling a sense of injustice” (5%), and “smoking tobacco” (5%). Therefore, when these reactions are reported in a certain section (e.g., “appealing to God” in the “Emotional Distress” section), it does not imply they were not also associated with other peritraumatic groups.

### Emotional Distress

The majority of participants mentioned fear as the predominant emotion experienced during the earthquakes. However, fear for the safety of others was reported by more participants (78% of the total sample) than fear for one’s own safety (70% of the total sample). Fear was conceptualized by participants as a social emotion often held in relation to significant others, especially family members not physically present at the scene. The first actions and thoughts of participants were often social in nature, with participants either calling by phone or actively going to search for significant others. Even when in extreme danger or distress, the thoughts of some participants were directed toward others. One participant who had remained stuck under debris described how:

I remember that I was there in the middle of all this debris and I thought, now I will die, and my family will die in another place, and I will die here on my own, I thought of my nephews, I thought of the people in my family, the people that I love that maybe were dying far away from me [cries]. [Female, 60 years]

Following fear for others, fear for one’s own safety was the most commonly reported emotion. Fear was often described as an overwhelming emotion, experienced in a “pure”^4^ form, absorbing all other cognitive and emotional resources by completely occupying one’s mental space. Participants described how their brains, used as a symbol for rationality, were emptied, zeroed, or blocked by fear. Participants variously described fear as clinging to them, taking control of them, or possessing them.

My first emotion was fear, an incredibly strong fear, a terrible fear, maybe the worst fear that I have ever experienced in my life, so much fear, terror, I was terrified, you know like when you go and watch an horror movie that you are not used to watching and you are scared even of small things like the sound of wind through the trees, it was pure terror, it was unbelievable. [Male, 52 years]

Fear was at times associated with participants holding catastrophic beliefs. These ranged from thinking that everyone they knew was dying or was going to die, that the earth was going to split under their feet and swallow them, or that the apocalypse was taking place.

I was terrified that the earth was going to split and open up, and that we were all going to be swallowed up by it, and I kept crying. [Female, 18 years]

Fear was associated with thoughts of death. Thoughts of dying and the possibility of experiencing a painful death were most fear-provoking and distressing to participants. When faced with the possibility of death, some participants described appealing to God or other religious figures. Religious figures were evoked generally to plead for survival but, in some cases, also for comfort when getting ready to die or when praying for a quick and painless death. An elderly woman recalled how:

I thought that now the ground will open up and I will go down, I was resigned to death, I made the sign of the cross and I waited for death [. . . ]“for all my sins” I said “please help me”, God will welcome me. [Female, 74 years]

Behaviorally, the emotion of fear was often associated with flight responses. A considerable number of participants described experiencing an urge to flee during the earthquake shocks. This instinct was generally triggered by the feeling of being trapped when inside built structures. It was described as a sudden, rushed, and not necessarily reasoned reaction, such as participants taking the stairs without checking they were intact. Houses and internal structures were generally conceptualized as spaces of risk one had to flee rather than as safe and solid refuges inside which one felt protected. Some houses were perceived as being so unsafe that a number of participants jumped from windows to get out of them.

Another commonly reported negative emotion was anxiety, often connected to feeling in a state of uncertainty. In addition, some participants reported having anxious thoughts concerning their future, such as what was going to happen to their lives and how they were going to continue living without family members, houses, and jobs.

Other emotions such as sadness, anger, guilt, and shame were also mentioned. Sadness, melancholy, crying, desperation, and emotional pain were feelings and behaviors associated with loss, often following the realization of the death of friends, family members, and acquaintances. This emotional pain was described as deep, excruciating, and insurmountable as well as being associated with feelings of unbridgeable void. The loss of people, in particular children, created gaping holes within the close-knit pre-earthquake social fabric, which participants felt could not be mended.

A number of participants also reported reacting to the events with anger, irritability, and frustration. Anger was often fueled by a perceived sense of injustice concerning the event. Participants often mentioned that what had happened was unfair, alluding to a tacit universal moral structure that had suddenly shattered. People reported asking themselves *why* such a thing had happened to them and what they had done to deserve so much pain.

### Action, Hyperfocus, and Emotion Regulation

Participants reported experiencing a considerable number of agency-driven reactions indicating orientation toward action, focus, and attempts at managing one’s emotions while in distress. Some participants reported quickly gaining full understanding of what was happening. Others described progressively initiating a process of making sense of the event and “putting things into focus” in the subsequent hours and days following a period of initial confusion.

The process of gaining awareness of what was happening was generally followed by a shift to actions oriented toward a goal. These actions were both directed toward the external environment, for example when providing support to others, and also toward oneself, such as by regulating one’s reactions. Participants reported “switching” to an operational mode by “unblocking” or “activating” oneself and “springing” into action. Some participants described this shift from confusion to action as that of resetting a frozen computer or phone.

It was only a moment of confusion, one second, then I saw the stones on the ground and I thought “shit, it’s the earthquake”, it was as if I had re-set my brain, and I went along with mechanical memory, I mean I had identified priorities 1, 2 and 3 and until I hadn’t completed all of these priorities I didn’t stop. [Female, 25 years]

A large number of different actions oriented toward a goal were described by most participants (72% of the total sample). The most commonly reported external action was that of providing both practical and emotional support to other people in need (64% of the total sample). Practical support ranged from offering food and water, giving people clothes and bedcovers, to providing first aid and rescuing people from under the debris. At times, participants reported putting their own safety at risk to help others. People often reported that what pushed them to provide practical support to members of their community as well as to strangers was the need to feel useful and take agency in relation to the situation together with an identification with the suffering of others during the event.

I put myself in the background in order to help others, I instinctively annihilated my ego, I considered the life of another person from Amatrice just as important as my own life. [Male, 28 years]

Participants also reported providing emotional support to others. This included calming and comforting others as well as being strong for people around them. One participant recalled his attempts to try and protect his elderly mother from the awareness of the loss of her town by telling her they were starring as background actors in a disaster movie and that everything she was witnessing was simply part of the movie set.

The act of providing emotional support was often associated with an attempt to put one’s emotions on hold to concentrate on the suffering of others. Participants reported engaging in various kinds of emotional labor to regulate, postpone, and control their emotions. The function of this emotional labor was often that of projecting and constructing an exterior impression of calm for others, sometimes actively “lying to oneself” due to internal turmoil. Participants conceptualized other survivors as open containers within which one could inject calmness, rationality, and tranquility. Emotions were described as communicable entities. Participants reported having been able to maintain a state of calmness and detachment by “freezing” and “turning off” their feelings. This emotional blunting was at times identified as a “defense mechanism” to handle particularly distressing scenes such as removing corpses from the debris.

I remember that every time that I heard of the death of someone I thought ok, in two or three days I will cry about the death of these people but now I have to try and help other people knowing that if I had let emotions take hold they might have stopped my action of helping others. [Male, 28 years]

Some participants also reported being strong, firm, and courageous, often to their surprise. A process that entailed an active search and buildup of internal strength and courage within oneself was at times described in terms of “working up courage.” One participant who had remained stuck under debris described how:

Generally I am a bit of a chicken, I faint, I am afraid of driving the car, I am afraid of everything, but when I was under there I felt such a strength, because you want to live and so you do everything in order to live. [Female, 60 years]

At the cognitive level, participants also described a particular state of enhanced focus on action. This was a state characterized by heightened levels of concentration and problem-solving, enhanced awareness and perceived rationality, mental lucidity and clarity, concrete thinking, and narrowing of attention to a specific aim. Participants recalled having few particular thoughts but rather being completely immersed in an action. The actions that participants were engaged in appeared to function as mental black holes, totally absorbing the cognitive capacities of the individual. Participants described this state of hyperfocus as similar to being in a state of trance.

My head was completely empty, I was only focused on acting [. . .], it’s not that while I was lifting the debris I was thinking about things, I didn’t think about anything, it was similar as when I go running and I focus on the run itself, on my breathing. [Male, 31 years]

### Cognitive Overload

The overwhelming intensity of thoughts, feelings, and sensory stimuli experienced during the traumatic event led some participants to enter a state of cognitive overload, a reaction consisting of moderate to severe disruption in how they processed information around them. This often led participants to feel confused during certain moments of the earthquakes. Participants described this reaction as feeling dazed or stunned and as not being able to fully take in, process, or understand what was happening. External and internal stimuli were described as being too intense and too fast-moving for cognitive capacities to keep up with them and assimilate them. The detachment from cognitive resources was described metaphorically by participants as being out of one’s mind, feeling absent, like a zombie, struck by lightning or drunk, and acting “without cognition.” This state was often heightened when participants reported being woken up by the strong shaking and failing to understand what was happening.

Cognitive capacities were conceptualized by participants as being a finite container that was overflowing with powerful internal and external stimuli, leading to confusion. Participants often compared their brains and minds with computers or mobile phones that were struggling to process the information received. Participants variously described their brain and minds as going into overload, haywire, or standby; as having to be reset or shut down; or as not connecting and stalling.

I had this phase of momentary blackout [. . .] I was blocked, I really couldn’t understand what I needed to do. In that moment everything is annihilated, it is as if you are a computer that has been reset, in that moment all the data in your brain have been zeroed by fear. [Female, 19 years]

The two core underlying triggers for this state of cognitive overload were identified by participants as feeling overwhelmed by emotions and feeling flooded by sensory stimuli, as if both the quantity and intensity of internal and external stimuli exceeded their psychological resources to process them. Two participants reported feeling as if this emotional load was making them “explode,” while others used the metaphors of being physically engulfed by an avalanche of emotions or “submerged” by emotions.

At the beginning you have too many emotions to manage and so your brain sort of goes in overdrive. [Female, 28 years]

Participants also reported that the amount and the intensity of sights, sounds, and smells contributed to the feeling of cognitive overload. Virtually every participant clearly recalled the darkness, the stench of gas, the taste of dust in one’s throat, the sound of people calling for help from under the debris, and the deafening rumble of the earthquake and of houses collapsing, together with the feeling of broken glass and sharp materials under one’s feet.

I remember this infernal heat, this heat that was suffocating me, mixed with the dust, and these deafening sounds of the ambulances, of the police cars, the sound of helicopters [. . .], this huge chaos, it disoriented me, it stunned me. [Female, 32 years]

Participants reported that thoughts appeared disorganized, racing, and disconnected one from another. Thoughts were described as possessing a materiality and a mass that caused them to “crowd,” “pile-up,” “cram,” and “condense” in one’s mind. In addition, thoughts acquired a “stickiness” that made them clump together and made it difficult for participants to distinguish one thought from the other. Interestingly, while some participants reported an overabundance of thoughts during cognitive overload, others also described an opposite state of thought vacuum and cognitive void where they reported experiencing no thoughts.

In the first moments I couldn’t divide different thoughts one from another, I couldn’t think rationally at only one thing at a time, in that moment they were all clumped together and I couldn’t manage to divide them. [Male, 20 years]

Some participants reported that while their basic functions such as perception kept working, they would fail to be integrated at a higher cognitive level with thoughts and beliefs. One participant described this as “seeing without understanding” and another participant as “the mind being outside of what the eye sees.”

I didn’t immediately gain awareness of the severity of the earthquake, I saw the stones of the school in the middle of the road but even there I didn’t really realize, I mean I saw them but it was as if I hadn’t seen them, it was a sort of seeing them but not thinking about it. [Female, 59 years]

This state of cognitive overload also led to disruptions in carrying out goal-oriented behaviors as participants reported not knowing what to do and how to react during and immediately after the events.

As with emotional distress, the initial phase of cognitive overload was, in some participants, followed by the ability to gather one’s cognitive resources and enter a more reactive and focused state. Others reported fluctuating between moments of cognitive overload and moments of rationality. Conversely, a minority of participants reported exiting this state of confusion and overload only days following the event.

### Dissociation

Participants reported experiencing a variety of peritraumatic dissociative reactions. These reactions clustered around three key phenomena: distortions in one’s sense of reality (i.e., derealization), distortions in one’s sense of self (i.e., depersonalization), and emotional numbness. The most commonly reported dissociative reaction was experiencing distortions in one’s sense of reality (51% of the entire sample). The disaster experience was permeated by a profound perception of “un-reality.” Some of the most common adjectives participants used to describe what they experienced were “surreal,” “absurd,” “impossible,” and “unbelievable” to indicate the disintegration of their perception of reality. The most widespread perception concerning derealization described by participants was the feeling of being in a dream during the earthquake events. This recurring comment concerning the dream-like quality of the experience might have also been partially due to most participant being woken from sleep by the earthquake. Participants also described feeling part of a movie, a fiction, a parallel reality, another dimension, or a videogame.

It felt as if I was in a dream, a thing that you don’t think it’s real, [. . .] I mean you were aware that it was all real but I had such a zeroing of emotions that I kept thinking “now I am going to wake up” I knew that it was real but at the emotional level it was as if it wasn’t real, a really strange thing. [Female, 23 years]

People reported struggling internally to determine whether what they were experiencing was real or the fruit of their fantasy. Reality was described as possessing a malleable and ambiguous quality as what they experienced deviated from their concept of normality so dramatically. The veil between reality and fantasy as well as between wakefulness and sleep had acquired a porous quality allowing one to blend into the other as participants described “losing touch with reality.” One participant described the urge to open the coffin of one of his deceased friends to actually make sure he had died and that he was not imagining everything. Participants proposed that derealization could have been a form of “self-defense” that their “brain” had actively conjured as an unconscious attempt to “reject” what was happening to them.

Together with a disintegration in the sense of reality, a smaller number of participants also reported disintegration in their usual sense of self and personhood (i.e., depersonalization). Participants reported not feeling like themselves or feeling outside of themselves. One participant described this sensation as being so acute that he started thinking he had disappeared or become invisible, a “ghost,” and touched himself to check he still existed. A further reaction associated with depersonalization was the feeling of being a spectator to what was happening or being “outside reality” rather than being directly involved in the experience.

It was as if I was outside of the world for a bit, it was me and only me, and the world was outside, as if there was no one else [. . .], I didn’t feel like myself. [Male, 19 years]

Participants who reported disruptions in sense of self and feeling “absent” often also described how at the emotional level they felt numb, apathetic, and empty. Participants reported a perception of emotional void; of having their affective resources completely depleted; of “feeling nothing,” feeling “emotionally blocked,” or unmoved. Participants described feeling their emotions being hollowed out like an “emptied carcass, only bones.” A participant described how, while his two children and partner were being extracted dead from the debris: *In these moments there are no emotions, it’s as if everything has stopped, inside of me*,

I felt it was useless to scream, it’s useless, I was a person of stone, blocked, inside you are blocked [. . .] I didn’t shed one tear, the feelings came after, in that moment there was nothing, [. . .] it’s unexplainable. [Female, 47 years]

### Mental Defeat and Loss of Control

Helplessness was a prevalent (60% of the total sample) peritraumatic response. The earthquake was often conceptualized as an entity that exceeded any human attempt to react to it. Indeed, while individuals reported losing their own sense of agency, they simultaneously projected a sense of all-powerful agency onto the earthquake itself. Many participants animated the earthquake constructing it as an active and intentional entity endowed with human-like traits such as cruelty, evil, ferocity, violence, and rage while also always remaining distinctly nonhuman in its omnipotent strength. Participants variously described the earthquake as a monster, a giant, a beast, and the devil and reported feeling chased by it or begging it to stop.

It felt as if we were inside the hands of a giant that did like this [makes shaking motion with hands], it moved us like a dice. [. . .]. [Female, 33 years]

This perceived loss of human agency was at times associated with a feeling of defeat, discouragement, and resignation as some participants reported losing all hope, feeling destroyed as a person, or “psychologically annihilated.” Some participants reported losing interest in whether they were going to live or die and surrendering to the event. This feeling of defeat was also associated with a perception of exhaustion as emotional, physical, and cognitive resources had been depleted, leaving participants feeling drained.

I felt weak in these moments, I had a feeling of powerlessness, like when you want to do something and you don’t manage to, as if you wanted to move a mountain, it’s impossible. [Male, 50 years]

Helplessness and the perceived loss of human agency also contributed to shifts in the perception of self and personhood during the earthquakes. Some participants reported feeling like a “pawn” or a “vegetable” at the mercy of nature. This sense of objectification was at times exacerbated to the point of participants perceiving themselves as being nothing or no one. Other participants described how the power of the earthquake made them feel like a “shit” or an “amoeba,” adding worthlessness to their sense of helplessness. Individuals felt hollowed out of their humanness as their life was perceived as becoming dependent on chance, miracles, luck, and nature rather than individual willpower. This perception was generally associated with feelings of being useless, insignificant, small, and defenseless as well as vulnerable, fragile, and lonely.

In that moment you realize you are a nullity, you realize that your life is worth nothing, it doesn’t matter how many people you saved or whether you built hospitals in all the world, it counts nothing, in that moment you are no-one, you are only something to get rid of, a pawn. [Female, 27 years]

### Immobility and Somatic Reactions

Participants also experienced a diverse range of psychosomatic reactions. The most commonly reported (37% of the total sample) psychosomatic reaction was physical immobility. Participants reported generally experiencing immobility during the moments corresponding to the earthquake shock, although some participants had also experienced the reaction when exposed to very distressing scenes. Physical immobility was often associated with feelings of loss of control over one’s own body. Participants generally described the sensation of having their entire body or specific body parts, usually legs and feet, blocked, heavy, stiff, rigid, paralyzed, or immobilized. They described feeling as if their body was “not responding” to intentional commands. Two participants described themselves as a “mummy” and as a “doll” respectively, inanimate objects unable to move autonomously.

During the moments when the shock was at its strongest, practically I felt that my legs were blocked, and therefore if I tried to take one step ahead I felt as if my legs were incredibly heavy, as if a leg weighted 200kg, I really couldn’t move, completely blocked. [Male, 25 years]

Some participants reported automatically feeling paralyzed, despite wanting to move. Among these participants, physical immobility was generally associated with feeling overwhelmed both emotionally and cognitively.

I remained still in my bed, clinging to my bed sheets, I remember hearing the sound of the walls crumbling and I understood that the house was breaking but I could not manage to comprehend the severity of the situation [. . .] in that moment I couldn’t do anything, I couldn’t manage to move, I didn’t manage to get up. [Female, 18 years]

Conversely, some participants reported a more deliberate instinct of trying to stay “still” due to feeling helpless. This appeared to be similar to a “playing dead” response. Indeed, as detailed in the mental defeat section, the earthquake was at times perceived as a possible predator, such as a monster or a beast. Some participants reported engaging in physical immobility as a survival strategy that could have been used against a living creature.

During the earthquake shock while me and my wife were hugging each other under the bed I kept telling her “be quiet be quiet be quiet” as if the earthquake went directly towards who screamed and instead the silence made you go unnoticed, as if, if he [the earthquake] didn’t hear us he would go somewhere else. [Male, 46 years]

People usually reported that they managed to exit this stage of immobility either during or immediately after the earthquake shock often due to other people around them encouraging them to “switch” to a reactive mode or because the acute phase of helplessness or emotional/cognitive overload had ended. Therefore, physical immobility was reported as being a transitory state usually lasting a few seconds or, less frequently, a few minutes. Some participants also reported that this moment of immobility was functional for them to orientate their attention to rationalize and understand what was happening.

Participants also reported psychosomatic reactions related to fear responses such as fainting, shaking, or losing one’s sense of appetite and thirst. In addition, in a minority of participants, a variety of psychosomatic reactions linked to immobility were reported such as not feeling pain (i.e., anesthesia), not being able to scream or shout (i.e., vocal suppression), and feeling cold.

While I was inside the debris as my house was collapsing, I didn’t feel any physical pain, this is adrenaline right? It felt as if I was falling in cotton-wool, but then when they brought me to the hospital I was covered in blood. [Female, 60 years]

### Positive Affect

A number of more positive emotions were also reported by participants. As with most other reactions, positive emotions were localized to specific moments of the traumatic events rather than generalized to the entire event. Hope was the most common positive emotion reported by participants (29% of the total sample). The earthquake events were conceptualized as spaces of intrinsic uncertainty and ambiguity, and while some participants filled this lack of information with catastrophic prospects and anxiety, others filled them with hope. Participants often reported hoping that people close to them had survived the earthquake, while some reported hoping that they were in a dream. Hope was generally described as being weak with participants speaking of “glimmers” or “strings” of hope that could be easily extinguished by the harshness of reality. Hope was often reported during moments when participants had not gained full awareness of the situation, although some participants reported “clinging” to hope despite clear contrary evidence.

A number of participants also reported transient feelings of joy and happiness in certain moments during the earthquake events. Seeing people being extracted alive from the debris or being reunified with family members were key moments when participants reported feeling joyful, happy, or relieved. These emotions were often reported as being circumscribed in time and “mixed” with a diverse range of other feelings and thoughts which often changed rapidly. In the midst of terror, fear, and anxiety, some participants were still able to identify moments of lightness. A participant who had lost her husband next to her described how she had felt comforted by the kindness of the medical personnel who had extracted her from the debris.

One moment we cried, one moment we laughed, and one moment we comforted each other, it’s a mix of feelings, it’s difficult to explain, it’s a jumble of sentiments, of thoughts. [Male, 25 years]

I remember when we saw all of our family members, or people that up to the day before you had never talked with and you would hug each other, there was this feeling of brotherhood, people that maybe before you didn’t even like, but seeing them there it was such a joy. [Female, 23 years]

## Discussion

This is the first study to explore naturalistically the lived experience of peritraumatic reactions in a large sample of individuals exposed to the same traumatic event. Our findings provide empirical support for the identification of peritraumatic dissociation, distress, immobility, and mental defeat in the quantitative literature. While some work has explored peritraumatic reactions qualitatively during distressing events, it has generally done so tangentially or in small samples (for tonic immobility, see Ayers, 2007; TeBockhorst et al., 2015, for peritraumatic dissociation, see Mattos et al., 2015). In addition, this is the first study to explore immobility and mental defeat during an earthquake. This shows that these two peritraumatic constructs, mostly conceptualized in relation to interpersonal violence, might be relevant to other traumas as well.

The accounts provide insight into an understudied peritraumatic reaction, that of cognitive overload, that is, a state of disruption in information processing mechanisms characterized by a perceived sense of confusion; a lack of integration of sensory-perceptual stimuli into higher cognition; and disorganized, overwhelming, and racing thoughts. Certain subcomponents of cognitive overload, such as confusion and disorientation, have been identified and explored in research (Dunmore et al., 2001; Kannis-Dymand et al., 2015; TeBockhorst et al., 2015) and are covered by certain items of the Peritraumatic Dissociative Experiences Questionnaire (Marmar et al., 1994) and the Data-Driven Processing Scale (Halligan et al., 2002). However, no systematic research had previously investigated the phenomenological characteristics of this construct in trauma-exposed populations.

Our results also highlighted the presence of a range of more adaptive and positive peritraumatic reactions such as hyperfocus on action and positive emotions such as hope, joy, and relief. These reactions have received virtually no attention in the peritraumatic literature, and its focus on more negative and dysfunctional reactions has meant that more normative aspects of trauma responses have been neglected (Bonanno, 2004). Survivors did not experience distress, dissociation, and helplessness passively but were able to respond and endeavor to manage these reactions through various coping mechanisms, such as emotional regulation and cognitive focus on goal-oriented actions. This could provide some explanation for the widespread psychological resilience shown by survivors following disasters (Bonanno et al., 2010) as these more neutral and adaptive reactions, and their consequent appraisal post-trauma, could play a protective role against post-trauma psychopathology. In addition, as Wilson et al. (2012) suggest, the identification of more neutral and adaptive reactions might represent a useful area of focus during trauma therapy to encourage the patient to build a more comprehensive and nuanced account of their trauma narrative.

Another core finding from the data cutting across most reactions was the inherently social dimension of the peritraumatic experience in the current sample, in that most participants reported noticing the reactions of others, fearing for others, and supporting others. While the findings are limited by possible social desirability biases, they are in line with findings from social psychology highlighting the cooperative, social, and nonselfish nature of most reactions during mass emergencies (Drury, 2018). However, a latent assumption underlying most of the peritraumatic literature is that individuals’ reactions are internal constructs uncorrelated and independent from the reactions of others who are present during the event. Across the 63 items of the six most widely used standard, quantitative peritraumatic measures,^5^ only 3 items acknowledge the possible presence of others during the traumatic event (e.g., Item 7 of the Peritraumatic Distress Inventory (Brunet et al., 2001) “I felt worried about the safety of others”). As many traumatic events are social in nature, future peritraumatic research should give more weight to the interactions between different individuals and pay more attention to the feeling of connectedness and relatedness to others that can be experienced during certain traumatic events.

Another theme cutting across most peritraumatic reactions concerns participants struggling to describe in their own words certain reactions they experienced, a phenomenon reported by more than half of the participants, with a quarter of the sample describing certain reactions as “strange.” In addition, one fifth of participants reported experiencing “mixed,” often contradictory, reactions at the same time, such as feeling hopeful but simultaneously sad. As a result of this, participants relied heavily on metaphors when describing their experience as ordinary language seemed to fail them in capturing its strangeness and complexity (van der Kolk & Fisler, 1995). This “indescribability” of certain traumatic constructs replicates findings from Černis et al. (2020) and provides empirical support for the linguistic work by Caruth (1996) concerning the unspeakability of trauma as a crisis in representation. These findings suggest that therapists might investigate the experience of peritraumatic reactions using means other than words, such as imagery, drawings, or body movement. In addition, future work might explore whether different degrees of “indescribability” of reactions are associated with different degrees of disruptions of higher order cognitive processing, and subsequently with different levels of PTSD.

Another novel contribution to the literature was the finding that participants reported fluctuating between a considerable variety of different peritraumatic reactions during the event (*M* = 21, range = 6–43). This highlights how the notion of a traumatic event might be best understood as an umbrella term containing within it many subevents with different peritraumatic characteristics (Marks et al., 2018). Participants reported “switching” out of initial negative peritraumatic reactions into more reactive modes, as well as fluctuating between different reactions and different degrees of the same reaction. The current quantitative methodology that requires participants to indicate the extent to which they experienced a certain reaction for the entire duration of the traumatic event might therefore be flawed. Future work should be more attentive to these fluctuations within and between reactions, especially because experimental work has shown that this fluctuation within peritraumatic reactions might be clinically meaningful, for example, in determining which moments of the traumatic event are encoded as intrusive memories (Chou et al., 2014). Furthermore, as participants often reported “switching” between different peritraumatic reactions during the course of the trauma, future research might attempt to identify the “switches” allowing people to move from negative peritraumatic reactions to more neutral and adaptive ones.

The current study has a number of limitations. These include the retrospective nature of the peritraumatic accounts, as the accuracy and consistency of traumatic memories is a subject of controversy (Brewin, 2018). While all peritraumatic recollections will be retrospective, future studies might attempt to collect data closer in time to the traumatic event. Another limitation concerns the possibility of social desirability biases skewing the reporting to socially acceptable reactions such as helping others and away from shame-provoking reactions such as selfish behavior. As Drury (2018) suggests, future work might attempt to diminish this bias by asking participants to describe the peritraumatic reactions observed in others rather than in oneself.

In addition, the open and exploratory method used in the current study, while allowing for narratives to arise organically and spontaneously, might have also led to the omission of certain reactions as participants only reported those that were salient and noteworthy for them. Future studies will be necessary to test whether the current reactions are generalizable to other survivors of other types of traumatic events in other cultural contexts. Furthermore, certain participants might differ in their ability to describe their own feelings and sensations, leading to variance in the amount and detail of reactions provided. Future work might attempt to control for this effect by measuring emotional granularity and interoceptive awareness among responders. In addition, although a number of precautions were taken such as ensuring not to inject any technical content into the interviews and having a second coder naïve to the field recode a subsection of the data, the awareness the authors had of previous work on peritraumatic reactions could have inadvertently biased the interpretation of the data toward preexisting conceptualizations of the reactions. Finally, the accounts reported represent the participants’ own subjective appraisal and recollection of their experience and might not necessarily coincide with data elicited through other types of measurement such as biological markers.

Besides having implications for mental health research and practice, our findings could also be useful for earthquake preparedness interventions because they provide detailed insight into how people may act, think, and feel during an earthquake (Joffe et al., 2019). They further highlight the role that members of the public can play in emergency response. As almost two thirds of participants reported trying to be useful, this should be factored into future disaster response plans (Ashkenazi & Hunt, 2019). Peritraumatic reactions, together with pretrauma and post-trauma factors, play a key role in the development of post-traumatic psychopathology and in influencing survivors’ wellbeing. The current article demonstrates the complexity, variety, and multifaceted nature of peritraumatic reactions during disaster. An improved understanding of their phenomenological characteristics is an important research and clinical priority.

## Supplemental Material

sj-pdf-1-qhr-10.1177_1049732320968791 – Supplemental material for Feelings, Thoughts, and Behaviors During Disaster Click here for additional data file. Supplemental material, sj-pdf-1-qhr-10.1177_1049732320968791 for Feelings, Thoughts, and Behaviors During Disaster by Alessandro Massazza, Chris R. Brewin and Helene Joffe in Qualitative Health Research

## Notes

1 Post-traumatic stress disorder (PTSD) is a psychiatric disorder that can develop following exposure to a traumatic event and is characterized by reexperiencing the event in the here and now through distressing memories and nightmares, avoiding reminders of the trauma, and by an ongoing sense of threat (World Health Organization, 2019).

2 This number was identified through a search on Google Scholar

3 The numbers provided in brackets do not necessarily indicate the actual prevalence of the reaction but simply how prevalent the reaction was in the spontaneous reports of participants.

4 When descriptions of the participants’ reactions are reported within quotation marks, it indicates that the exact wording of the participant was used.

5 Peritraumatic Dissociative Experiences Questionnaire, Peritraumatic Distress Questionnaire, Tonic Immobility Scale, Mental Defeat Questionnaire, Somatoform Dissociation Questionnaire-Peritraumatic, Data-Driven Processing Scale
