# Supplementary material for: Feelings, Thoughts, and Behaviors During Disaster
Source: Qual Health Res. 2020 Nov 23;31(2):323–37. doi: 10.1177/1049732320968791 (PMC7753093; doi:10.1177/1049732320968791)
Supplement: sj-pdf-1-qhr-10.1177_1049732320968791 – Supplemental material for Feelings, Thoughts, and Behaviors During Disaster [file sj-pdf-1-qhr-10.1177_1049732320968791.pdf]

Table A
Demographic statistics of participants

Variable	Percentage of total sample or total mean
**Gender and age**	
Male	46% (mean age = 44.23, range = 19-75)
Female	54% (mean age = 43.04, range = 18-74)
**Religion**	
Catholic	74%
Other	26%
**Educational level reached**	
Secondary school	51%
Middle school	26%
University degree	21%
Primary school	2%
